# Preterm Birth in Assisted Reproductive Technology: An Analysis of More Than 20,000 Singleton Newborns

**DOI:** 10.3389/fendo.2020.558819

**Published:** 2020-10-07

**Authors:** Zhiqin Bu, Jiaxin Zhang, Linli Hu, Yingpu Sun

**Affiliations:** Reproductive Medical Center, Henan Province Key Laboratory for Reproduction and Genetics, The First Affiliated Hospital of Zhengzhou University, Zhengzhou, China

**Keywords:** preterm birth, assisted reproductive technology, risk factors, maternal age, body mass index–BMI

## Abstract

**Background:**

Several studies have shown that newborns conceived through the ART procedures were associated with an increased incidence of preterm delivery compared to those conceived spontaneously regardless of the type of ART procedure. The aim of the study was to explore risk factors for preterm birth (PTB) in assisted reproductive technology.

**Method:**

In this single center retrospective cohort study, a total of 23,111 singleton newborns from January 2010 to December 2018 were included. The primary outcome in this study was preterm birth, defined as live birth before 37 weeks’ gestation.

**Result:**

In the 23,111 pregnancies, the incidence of PTB was 7.13%. In multivariate logistic regression analysis model, BMI was an independent predictor for PTB (OR = 1.05, 95% CI: 1.03–1.07; P = 0.00 in IVF/ICSI cycles; OR = 1.08, 95% CI: 1.03–1.12; P = 0.00 in IUI cycles). Also, another independent predictor in ART was male newborns (OR = 1.27, 95% CI: 1.14–1.41; P = 0.00 in IVF/ICSI cycles; OR = 1.60, 95% CI: 1.17–2.18; P = 0.00 in IUI cycles). In IVF/ICSI cycles, PTB was significantly higher in patients with advanced age (9.56% in ≥ 38 years old), and in patients with a history of cesarean section (10.39%). In addition, Blastocyst transfer, and frozen thawed transfer were also risk factor for PTB as compared with cleavage transfer, and fresh transfer, respectively. Moreover, in frozen thawed transfer cycles, stimulated protocol (estrogen-progesterone) increased PTB as compared with natural protocol (OR = 1.33, 95% CI: 1.12–1.59; P = 0.00). This situation was similar in IUI cycles as stimulated protocol also increased PTB when compared with natural protocol (OR = 1.72, 95% CI: 1.19–2.48; P = 0.00).

**Conclusion:**

Body mass index (BMI), delivery with male newborn, as well as stimulated protocols, were independent risk factors for PTB in both IVF/ICSI and IUI treatment cycles. In IVF/ICSI cycles, independent risk factors also included maternal, history of cesarean section, frozen thawed transfer, and blastocyst transfer.

## Introduction

Recently, there are more and more infertile couples getting their own babies by assisted reproductive technology (ART). Even though many progress have been made regarding to live birth rate, the safety of this technology is still a huge concern for both patients and physicians. Preterm birth (PTB), which is defined by the World Health Organization as delivery before 37 completed weeks of gestation, is one of the leading causes for infant morbidity and mortality in many countries (1).

Several studies have shown that newborns conceived through the ART procedures were associated with an increased incidence of preterm delivery compared to those conceived spontaneously regardless of the type of ART procedure (2–4). According to previous studies, several possible factors such as embryo culture medium, *in vitro* culture to blastocyst stage, as well as embryo cryopreservation, might be related to PTB in couples treating with ART. In 2013, a study reported that extended culture has previously been held responsible for a higher risk of preterm delivery (5). However, several other reports are not in line with this study (3, 6, 7). More recently, another large sample retrospective analysis involved 67,147 *in vitro* fertilization/intracytoplasmic sperm injection (IVF/ICSI) cycles reporting there was no significant difference in the risk of PTB after blastocyst stage embryo transfer compared with cleavage stage group (8). Meanwhile, data also showed that singleton pregnancies after transfer of frozen thawed embryos were associated with a lower preterm birth when compared with those after fresh embryo transfers (2).

It is not surprised to see conflicting results from these cohort studies, as not only large sample size is needed, multivariate analysis is also necessary. Thus, we included all singleton newborns conceived by ART in our center since 2010, to explore possible risk factors for PTB in this multivariate analysis.

## Materials and Methods

This retrospective cohort study has been approved by the Institutional Review Board (IRB) of First Affiliated Hospital of Zhengzhou University. As a routine practice, we have recorded each patient’s basic parameters and clinical treatment outcomes in Clinical Reproductive Medicine Management System/Electronic Medical Record Cohort Database (CCRM/EMRCD) since 2009. Data in this study were from CCRM/EMRCD in Reproductive Medical Center, First Affiliated Hospital of Zhengzhou University. Written informed consent was obtained from all patients before treatment.

Inclusion criteria were patients undergoing assisted reproductive technology from January 2010 to December 2018. Intrauterine insemination (IUI) cycles included artificial insemination with husband sperm (AIH) and artificial insemination with donor sperm (AID); IVF/ICSI cycles including fresh and frozen thawed embryos transfer with both cleavage stage embryo and blastocyst stage embryo. Only patients with singleton live birth were included. Exclusion criteria were: patients with uterus malformation, pre-implantation genetic testing (PGT) cycles, as well as oocyte donation cycles. In addition, patients with singleton delivery, but had a history of artificial multiple pregnancy reduction/vanish twin were also excluded.

The primary outcome in this study was preterm birth, defined as live birth before 37 weeks’ gestation. Patient basic parameters included maternal age, body mass index (BMI), main diagnosis for infertility, stimulation/non-stimulation for ART, ovarian stimulation protocols, and number/type of embryos transferred. Since women smoking rate in China is very low, and none of these patients included reported a history of smoking, the association between smoking and PTB was not evaluated in the current study.

### Statistical Analysis

First, patents were divided into different groups according to basic parameters, and PTB rate was compared among these groups in both IVF/ICSI and IUI cycles using Chi-square test. Multivariate logistic regression was performed to evaluate the association between the variables and PTB. A *P* value < 0.05 was considered statistically significant. All data were included into SPSS (Statistical Package for Social Science, SPSS Inc, Chicago, IL) 21.0 for analysis.

## Results

In the 23,111 pregnancies, the incidence of PTB was 7.13% (1,647of 23,111). The incidence of PTB in IVF/ICSI and IUI cycles was 7.43% (1,469 of 19,781) and 5.35% (178 of 3,330), respectively.

For IVF/ICSI, the incidence of PTB was only no different between GnRH agonist and GnRH antagonist cycles (6.29% vs 7.84%; P = 0.27). However, PTB was different in patients according to Maternal age, Body mass index (BMI), Infertility diagnosis (primary/secondary infertility), History of cesarean section, Type of embryo transfer (fresh/frozen thawed), Stage of embryo (cleavage/blastocyst), Number of embryos transferred (**Table 1**). In addition, PTB in patients with a male newborn was significantly higher than that in patients delivered with a female newborn (8.20% vs 6.60%; P = 0.00). Moreover, in frozen thawed embryo transfer cycles, PTB was different in patients with estrogen-progesterone protocol and natural cycle for endometrial preparation (9.54% vs 7.33%; P = 0.00).

**Table 1:** Preterm birth in IVF/ICSI treatment cycles.

	Preterm birth rate	P
Age (years)		0.000
≤28	6.55% (480/7332)	
29 – 37	7.72% (843/10922)	
≥38	9.56% (146/1527)	
BMI (kg/m^2^)		0.000
≤18.5	6.60% (90/1363)	
18.6-24.9	6.91% (987/14285)	
≥25	9.46% (392/4143)	
Infertility diagnosis		0.000
Primary infertility	6.56% (650/9903)	
Secondary infertility	8.29% (819/9878)	
History of cesarean section		0.000
Yes	10.39% (186/1790)	
No	7.13% (1283/17991)	
Type of transfer		0.000
Fresh embryo	6.33% (716/11314)	
Thawed embryo	8.89% (753/8467)	
Stage of embryo		0.000
Cleavage stage	6.93% (1,038/14971)	
Blastocyst stage	8.96% (431/4810)	
Protocols-Fresh cycle		0.270
GnRH agonist	6.29% (692/11,008)	
GnRH antagonist, others	7.84% (24/306)	
Protocols-frozen cycle		0.001
Natural cycle	7.33% (181/2,469)	
Estrogen-progesterone	9.54% (572/5,998)	
No. of embryos transferred		0.005
1	8.30% (357/4,300)	
2	7.05% (997/14147)	
3	8.62% (115/1334)	
Sex of newborn		0.000
Male	8.20% (838/10225)	
Female	6.60% (631/9556)	

IVF, in vitro fertilization; ICSI, intracytoplasmic sperm injection; BMI, body mass index; GnRH, gonadotrophin-releasing hormone.

As shown in **Table 2**, PTB also varied between IUI patients with different maternal age, BMI, Infertility diagnosis, History of cesarean section. However, only the difference of PTB in BMI groups reached statistical significance (8.31%, 4.59%, and 3.62%; P = 0.00). Interestingly, PTB was also found to be different in patients with stimulation cycle and natural cycle (6.56% vs 3.38%; P = 0.00), in patients delivered with male and female newborns (6.49% vs 4.20%; P = 0.00). Moreover, PTB was higher in AIH cycles when compared with that in AID cycles (6.73% vs 4.08%; P = 0.00).

**Table 2:** Preterm birth in IUI treatment cycles.

	Preterm birth rate	P
Age (years)		0.141
≤ 28	5.16% (105/2035)	
29–37	5.37% (66/1230)	
≥ 38	10.77% (7/65)	
BMI (kg/m^2^)		0.000
≤ 18.5	3.62% (10/276)	
18.6–24.9	4.59% (104/2267)	
≥ 25	8.13% (64/787)	
Infertility diagnosis		0.700
Primary infertility	5.44% (129/2371)	
Secondary infertility	5.11% (49/959)	
History of cesarean section		0.752
Yes	5.79% (14/242)	
No	5.31% (164/3088)	
Protocols-IUI cycle		0.000
Natural cycle	3.38% (43/1273)	
Stimulation cycle	6.56% (135/2057)	
Sperm source		0.001
Husband	6.73% (107/1591)	
Donor	4.08% (71/1739)	
Sex of newborn		0.003
Male	6.49% (108/1664)	
Female	4.20% (70/1666)	

IUI, intrauterine insemination; BMI, body mass index.

In **Table 3**, all PTB-related factors as shown above were re-analyzed at the same time in a multivariate logistic regression analysis model. In both IVF/ICSI and IUI cycles, BMI was an independent predictor for PTB (OR = 1.05, 95% CI: 1.03–1.07; P = 0.00 in IVF/ICSI cycles; OR = 1.08, 95% CI: 1.03–1.12; P = 0.00 in IUI cycles). Also, another independent predictor in ART treatment was sex of newborns (OR = 1.27, 95% CI: 1.14–1.41; P = 0.00 in IVF/ICSI cycles; OR = 1.60, 95% CI: 1.17–2.18; P = 0.00 in IUI cycles). In addition, Blastocyst transfer, and frozen thawed transfer were also risk factor for PTB as compared with cleavage transfer, and fresh transfer, respectively. Moreover, in frozen thawed transfer cycles, stimulated protocol (estrogen-progesterone) increased PTB as compared with natural protocol (OR = 1.33, 95% CI: 1.12–1.59; P = 0.00). This situation was similar in IUI cycles as stimulated protocol also increased PTB when compared with natural protocol (OR = 1.72, 95% CI: 1.19–2.48; P = 0.00).

**Table 3:** Factors associated with PTB by logistic regression analysis.

	IVF/ICSI		IUI	
	Adjusted OR (95% CI)	*P* value	Adjusted OR (95% CI)	*P* value
Maternal age	1.02 (1.01–1.04)	0.00	1.02 (0.98–1.06)	0.34
BMI (kg/m^2^)	1.05 (1.03–1.07)	0.00	1.08 (1.03–1.12)	0.00
History of cesarean section (yes/no)	1.32 (1.11–1.56)	0.00	1.05 (0.58–1.89)	0.87
Sex of newborn (male/female)	1.27 (1.14–1.41)	0.00	1.60 (1.17–2.18)	0.00
Type of transfer (thaw/fresh)	1.36 (1.22–1.52)	0.00	–	–
Stage of embryo (blastocyst/cleavage)	1.20 (1.06–1.35)	0.00	–	–
Type of cycle (stimulated/natural)	–	–	1.72 (1.19–2.48)	0.00
Sperm source (husband/donor)	–	–	1.30 (0.93–1.81)	0.13
Thaw (EP/natural)^*^	1.33 (1.12–1.59)	0.00

IVF, in vitro fertilization; ICSI, intracytoplasmic sperm injection; IUI, intrauterine insemination; OR, odds ratio; CI, confidential interval.

^*^Only in frozen-thawed cycles.

## Discussion

PTB is a complicated syndrome that increases morbidity and mortality for mother and infant. According to several previous studies, singletons born after IVF/ICSI treatment have an increased risk of PTB compared to those spontaneously conceived (3). Since IVF/ICSI, as well as another artificial reproductive technology IUI, involve several *in vitro* manipulation procedures of gametes/embryos, it is necessary to explore such risk factors that related to PTB in newborns conceived by ART, and to inform both physicians and patients for choice making.

First, the current study showed that the incidence of PTB in IVF/ICSI was 7.43%, which was significantly higher than 5.35% from IUI cycles. According to data from a Meta analysis including PTB from both IVF/ICSI and spontaneously conceived babies, PTB were 10.1% (IVF/ICSI treatment) and 5.5% (spontaneously conceived), respectively (3). Results from our study were not surprising, and consist with previous literature (3). IUI, which only involves sperm preparation and/or ovulation induction, is more similar with natural conception. The difference of PTB between IVF/ICSI and IUI cycles highlighted the impact of embryo *in vitro* manipulation on PTB.

As for patients’ basic characteristics, we explored the impact of maternal age, history of cesarean section, and BMI on PTB in both IVF/ICSI and IUI cycles. In summary, our data showed that women with advanced maternal age, a history of cesarean section, and obesity were at great risk of PTB, which was consistent with previous findings (9, 10). These three cofounders always interact with each other, and can impact PTB together. As the rates of pre-existing diabetes, medical invasive procedure, placenta previa, and obesity were all more common in aged mothers, a recent large sample size cohort study with 165,282 births showed that rate of preterm birth <37 weeks was lowest in the 30 to 34 years old group (5.7%) and highest in women over 40 years (7.8%) (9). In addition, it was also shown that there was a J-shaped association between the absolute risk of preterm birth and increasing BMI category, as obesity was also showed to be associated with increased risks including pre-eclampsia, gestational diabetes, admission to intensive care, and caesarean section (11, 12).

Another main finding from the current study was that both frozen thawed embryo transfer and blastocyst transfer significantly increased the chance of PTB even after adjusting maternal age, BMI, and other confounders. The comparison of the risk of PTB between frozen and fresh embryo transfer yielded conflicting results. Some studies showed that cryopreservation of embryos can influence PTB (13), while others did not show any difference regarding PTB between frozen and fresh embryo transfers (14, 15).

In addition, even PTB was observed to be higher in blastocyst transfer as compared with cleavage transfer in most previous studies (16–18), while other studies still showed conflicting results (19). We believe that several reasons may explain the controversy between previous studies: First, most studies, including ours, did not cover all possible PTB related confounders in analysis. In addition, large sample cohort studies always included data from multi-centers, or from a long period of time, in which the methods of embryo cryopreservation, embryo thawing, *in vitro* culture system and medium changed dramatically. Data in the current study were from a single center, and only included frozen embryos using vitrification technology; however, embryo culture environment did change during the past 9 years in our center.

Another interesting, but also important, result from our data was that PTB rate in women born with a male was much higher than that in women pregnant with a female. This phenomenon has also been shown in previous population-scale data. Possible mechanisms for this disparity are not clear, but may related to intrauterine inflammation or infection-response associated with higher concentrations of pro-inflammatory markers in male infants, and also variability in selection pressures on females or males to survive to term gestation (20). This also warns us that the situation of both mothers and fetus after ART treatment, which are always lacking in previous analysis, should be considered in future cohort studies.

In addition, several studies have focused on the impact of ovarian stimulation on ART outcome as compared with natural cycles (21–24). However, unlike two previous studies in which no difference was found regarding perinatal outcomes between stimulated and unstimulated cycles (21, 22), our data demonstrated that PTB rate in stimulated cycles was significantly higher in both IVF/ICSI and IUI cycles. Nevertheless, we should be caution when interpreting this result. For ovulation induction in IUI cycles and endometrial preparation in frozen embryo transfer cycles, stimulated protocol was more common in patients with ovulation disorder, such as PCOS. Thus, prospective randomized studies are needed to clarify this issue in the future.

The strength of our study is that the sample size is large, and all treatment cycles are from the same center. In addition, we tried our best to include as many PTB related factors as we can to reach accurate conclusions. However, as a retrospective studies, the current one indeed also has several limitations. Our study included cycles from a 9-year period, during which the technology and *in vitro* culture environment have developed. Meanwhile, we cannot separate spontaneously preterm birth (SPTB) and iatrogenic preterm birth (IPTB), in fact the risk factors and prognosis differ markedly between these two subtypes. In addition, information regarding maternal characteristics, such as the causes of infertility, the history of preterm birth, genital tract infection, pregnancy complications, abnormal placenta, which may be important with preterm birth were not available.

Taken together, results from our study showed that PTB was higher in IVF/ICSI cycles as compared with IUI cycles. BMI, delivery with male newborn, and stimulated protocols were independent risk factors for PTB in both IVF/ICSI and IUI treatment cycles. In addition, maternal age, history of cesarean section, frozen embryo transfer, and blastocyst embryo transfer in IVF/ICSI cycles also increased PTB. However, since our study was based on retrospective collected data with several limitations, further high-quality studies are needed to reach a more convincing conclusion regarding risk factors for PTB in ART.

## Data Availability Statement

The raw data supporting the conclusions of this article will be made available by the authors, without undue reservation.

## Ethics Statement

The studies involving human participants were reviewed and approved by Institutional Review Board (IRB) of First Affiliated Hospital of Zhengzhou University. The patients/participants provided their written informed consent to participate in this study.

## Author Contributions

ZB and JZ contributed to execution of the study, completed the data analysis and wrote the article. LH and YS conceived the study design, contributed to interpretation of the data, critically revised the article and approved the final version.

## Funding

This study was supported by National Key R&D Program of China 2019YFA0110900 to YS; National Natural Science Foundation of China 81801448 to ZB.

## Conflict of Interest

The authors declare that the research was conducted in the absence of any commercial or financial relationships that could be construed as a potential conflict of interest.
